# Nanostructuration of SmCo_5_ Magnets by Spark Plasma Sintering: Optimization of Magnetic Properties

**DOI:** 10.3390/ma19194260

**Published:** 2026-10-08

**Authors:** Nicolas Albar, Frédéric Adamski, Sulivan Kuttler, Pierre Sallot, Claude Estournès

**Affiliations:** 1 Univ. Toulouse, Toulouse INP, CNRS, CIRIMAT, 118 Route de Narbonne, CEDEX 9, 31062 Toulouse, France; nicolas.albar@utoulouse.fr; 2 Materials and Processes, Safran Tech, Rue des Jeunes Bois, Châteaufort, 78114 Magny-Les-Hameaux, France; frederic.adamski@safrangroup.com (F.A.); sulivan.kuttler@safrangroup.com (S.K.); pierre.sallot@safrangroup.com (P.S.)

**Keywords:** magnetic materials, spark plasma sintering (SPS), microstructure, electron backscattered diffraction (EBSD), high power density

## Abstract

In the context of current climate issues and environmental constraints, green and renewable energies have experienced considerable growth in recent decades. Thus, there has been a renewed interest in the development of electric machines. To achieve this, the optimization of mass saving, volume and magnetic performance in the development of these machines must accelerate and be implemented industrially, particularly in the aeronautical field. The aircraft of the future will therefore require the use of electric machines with very high power densities and efficiencies. To achieve these objectives, one of the essential components to improve is the permanent magnet, and in particular its magnetic properties (increases in coercive field, residual magnetization and specific energy density). These magnets are likely to operate at temperatures above 200 °C. In this context, hard ferromagnetic magnets seem to be the most appropriate. SmCo_5_ magnets have a Curie temperature between 720 and 920 °C. In this work we show the impact of grinding time on the magnetic properties of these SPS-treated magnets. This method allows the sintering SmCo_5_ magnets while limiting their oxidation and their grain growth. By optimizing the grinding parameters, we obtain magnets with a coercive field of the order of 18,800 Oe. The novelty of this study lies in the optimization of EBSD (Electron Backscatter Diffraction) image analysis using the NLPAR (Non-Local Pattern Averaging Reconstruction) method, combined with advanced image correlation techniques. This approach enabled the precise determination of a grain size distribution centered around 50 nm, offering new perspectives on microstructural control and the enhancement of magnetic performance in SmCo_5_ magnets.

## 1. Introduction

With the application of green and renewable energies in future vehicles, these vehicles’ electrical systems will require machines with higher power density and higher efficiency. A way to increase compactness while maintaining or even improving machine efficiency is to increase magnet performance. Permanent magnets are key materials in electrical applications such as car engines and electrical generators in wind turbines [1]. The use of these magnets currently offers the best power density in synchronous machines in comparison with other electrical machines such as DC motors or asynchronous motors [2]. However, even in rotors with magnet structures, the operating temperature must be taken into account. The rotor is heated both by its proximity to the stator and by its internal losses, particularly those occurring in the magnets. Two high-performance magnet types, based on NdFeB and SmCo alloys, are commonly implemented in synchronous machine rotors, as they exhibit the highest power densities reported in the literature, with respective energy densities between 135 and 310 kJ/m^3^ [3,4,5]. SmCo-type magnets have a Curie temperature in the range of 720 to 920 °C [6,7,8], which is significantly higher than that of NdFeB magnets, which is around 320 °C. This is a critical parameter with respect to the operating temperature of the electric machine and the resulting residual induction at that temperature. It therefore enables the appropriate type of magnet to be selected for a given application. Indeed, the performance of NdFeB magnets degrades significantly at high temperatures compared to that of SmCo magnets, and the energy density of the magnets strongly depends on of the rotor operating temperature. Another parameter is the maximal operating magnet temperature before permanent partial demagnetization. This temperature is related to the Curie temperature, where the magnet is completely demagnetized, i.e., between 300 and 400 °C for NdFeB and between 700 and 900 °C for SmCo [6,7,8]. Increases in temperature involve a decrease in coercive field [9,10]. The maximal operating temperature is around 200 °C for NdFeB and 300 °C for SmCo, which is one of the limitations of using NdFeB for critical applications such as in aeronautical systems. In addition, these SmCo magnets are also more resistant than NdFeB to oxidation [11,12,13]. To optimize the performance of magnets, two strategies can be implemented. First, the size of the magnetic domains can be optimized by carefully selecting the appropriate particle size of SmCo_5_ powder. The second approach involves maximizing the energy density ((BH)_max_) of the magnet by increasing the residual magnetization value. Regarding the first approach, the powder should be selected to achieve a relative density as close to 100% as possible, while also maximizing the coercive field (Hc). It has been established in the literature that, as the grain size in magnetic materials decreases, Bloch walls gradually disappear until a critical size (Ds) is reached—equivalent to that of a magnetic monodomain. As illustrated in Figure 1 [14], when reducing grain size, the coercive field increases. When the grain size reaches Ds, the coercive field (Hc) can achieve its optimal value [8]. However, if the grain size decreases below Ds, the coercive field also decreases. Consequently, the maximum that Hc can reach is highly dependent on the grain size distribution within the magnet. As the grain size continues to decrease, thermal agitation energy eventually exceeds the magnetic anisotropy energy. This defines a second critical grain size, Dp, at which a superparamagnetic regime is reached. Zhang et al. demonstrated that at a particle size of 40 nm, a Hc value of 28,500 Oe can be achieved [15].

The second strategy to optimize magnet performance focuses on increasing the residual magnetization (Mr) value, which directly impacts the maximum energy density (BH)_max_ [16]. Previous work has already explored orienting powder particles during mold preparation to align the easy magnetization axes in a single direction. This alignment ensures that, once the magnet reaches saturation (M_s_), M_r_ remains close to M_s_ [17]. The objective of the present work is to investigate the sintering of SmCo_5_ magnets using Spark Plasma Sintering (SPS) in order to establish a robust processing protocol that enables the production of specimens with enhanced power densities as well as the highest possible coercive field. Our work focuses on powder optimization properties, especially investigating the impact of grinding on the magnetic characteristics of the SPS-treated samples. The image analysis method presented allows for the definition of grain size distributions on the scale of tens of nanometers using the Electron Backscatter Diffraction (EBSD) method. Given that, the goal is to control the microstructure to achieve a final grain size as close as possible to that of a single magnetic domain; this is why Spark Plasma Sintering (SPS) emerges as a highly promising process. The combination of uniaxial pressure and Joule heating enables lower sintering temperatures while effectively limiting grain growth during the process. As observed in the fabrication of NdFeB magnets, Nd-rich phases promote sintering by forming a liquid phase at grain boundaries [16]. In the case of SmCo magnets, these phases also help to minimize samarium evaporation, thereby preserving the desired stoichiometry [18,19]. Importantly, these phases remain magnetic and contribute to the overall magnetization of the material. However, preventing samarium oxidation during sintering is critical, as the non-magnetic Sm_2_O_3_ phase can act as a nucleation site for reverse magnetic domains, ultimately reducing the coercive field [13]. For this reason, all handling and processing were conducted under an inert atmosphere. In bulk materials, grain sizes typically range in the tens of nanometers. Conventional methods for determining grain size distribution lack the precision required to characterize these samples accurately. Therefore, optimizing images obtained through the NLPAR method detailed below is essential.

## 2. Experimental Section

This study used commercially available SmCo_5_ powder sourced from American Elements, USA with a purity of 99.9% rare earth and agglomerate sizes below 150 µm. The powder had a density is 8.51 g/cm^3^, as measured by an AccuPyc II 1340 helium pycnometry from Micromeritics, USA. It was ground in a tungsten carbide grinding bowl in a PM100 Retsch planetary miller, Germany, under argon—to avoid samarium oxidation—for durations of 0 h, 1 h, 2 h and 4 h at 400 rpm. The ball-to-powder weight ratio was maintained at 10:1 with tungsten carbide balls. For the sintering experiments, a WC-Co die (8 mm inner diameter) was filled with 0.85 g of SmCo_5_ powder necessary to obtain a 2 mm high dense cylindrical sample. To avoid direct contact between the die, punches and powder, a 0.2 mm graphite foil (PERMAFOIL^®^Toyo Tanso, France) was placed between the powder and each punch, as well as vertically between the inner wall of the die and the powder. Sintering experiments were performed with SPS apparatus (FUJI 632Lx from Fuji Electronic Industrial CO., Japan) using pulsed DC current. The experiments were carried out at the Plateforme Nationale CNRS de Frittage Flash at the Université de Toulouse. Temperature was monitored using a K-type thermocouple inserted into a hole located midway along the outer surface of the die. The optimized sintering cycle consisted of a dwell temperature of 700 °C for 5 min, with a heating ramp rate of 100 °C/min. A pressure of 500 MPa was applied at room temperature before the temperature increase. An additional experiment was performed at 700 °C with a dwell time of 20 min on the powder that had been ground for 4 h, at the same pressure of 500 MPa. The samples were named BX-FY, where X is the milling time and Y the sintering time. The sintering was carried out under a vacuum to limit the oxidation of the samarium. Density measurements were further performed on sintered specimens by Archimedes’ method with a Sartorius balance MSE224S-YDK03, Germany. The crystalline phases were analyzed using X-ray diffraction (XRD) with a Bruker D4 Endeavor diffractometer, Germany (Cu-Kα at a wavelength of 0.15406 nm) and a Malvern Panalytical X’Pert Pro diffractometer, Netherlands (with Co anode, wavelength 0.17892 nm). Magnetic measurements were performed at 300 K with a Quantum Design Versalab vibrating sample magnetometer, USA (VSM) capable of generating a magnetic field of up to 3 T and an extraction magnetometer developed at the Néel institute, with a magnetic field of 9 T. For microstructural characterization, the samples were embedded with a conductive thermopolymerizable PolyFast resin using a Struers CitoPress-20 coating machine, Denmark with a diameter of 30 mm. The embedded sections were subsequently polished for the Electron Backscatter Diffraction (EBSD) analysis, with a final step involving chemical and vibrational polishing using colloidal silica with a particle size of 0.02 μm. The EBSD measurements were performed using a Zeiss CrossBeam 550 scanning electron microscope (SEM-FEG), Germany, operated at an accelerating voltage of 15 kV and a probe current of 5 nA. An Oxford Instruments Symmetry S3 detector, UK, was utilized, coupled with Aztec v.6.2 acquisition software set to “High-Sensitivity” mode. This setup enabled an acquisition rate of 200 Hz for 156 × 126 pixels^2^ electron backscatter patterns (EBSPs). The resulting maps, including EBSPs, were saved in .h5oina format for subsequent reprocessing. The EBSD maps with areas of 5.6 × 4.2 µm^2^ were acquired with a step size of 25 nm for the three ground samples, whereas the raw material map used a step size of 1 µm and an area of 320 × 240 µm^2^. Initially, indexation was performed using the classic Hough transform method and the “Optimized Band Detection” option within the Aztec software. However, the orientation maps of the three ground samples were not analyzable due to an indexation rate of around 50%, attributed to the extremely small grain size. To improve these results, two complementary methods were implemented. The first enhancement method implemented was Non-Local Pattern Averaging Reindexing (NLPAR), designed to improve noisy EBSP data and boost indexing success. This algorithm uses a non-local smoothing kernel, where weights are determined by the exponential decay of the Euclidean distance between patterns [20]. Implemented in the python library pyEBSDIndex v.0.3.7 [21], NLPAR made diffraction bands more visible, though the traditional Hough transform remained insufficient for correct indexing. Additionally, dictionary indexing was applied, replacing the classical Hough method to improve the indexing rate. This method involves correlating experimental EBSPs with theoretical and simulated EBSPs [22]. To simulate all possible orientation snapshots for the crystalline lattice geometry, a master pattern encompassing diffraction in all spatial directions was generated using the open-source software EMSoft (v.5.03, [23]). Using the SmCo_5_ crystal geometry described in [24], that was able to produce optimal Hough indexing in Aztec software, a master pattern for diffraction under a 15 kV electron beam (Figure 2) was generated. The pattern matching algorithm was then executed using the python library Kikuchipy (v.0.10) [25] through the following steps:

1. Definition of a matrix R with all possible crystal orientations at a 1.5° resolution.
2. Simulation of EBSP dictionaries from orientations in matrix R, using the master pattern and detector geometry (size, pattern center, tilt).
3. Calculation of cross-correlation between simulated and experimental images. The simulated image with the highest Normalized Cross-Correlation (NCC) was retained as the orientation. The EBSD map was indexed with a discretized step size of 1.5° on Euler angles.
4. Optimization of NCC at each point using a Nelder–Mead algorithm, adjusting the crystal orientation and projection center with simulations around the previously estimated orientation, using a 0.001 convergence criterion on NCC.

Figure 3 presents two experimental EBSPs, their NLPAR transformation, and the simulated EBSP showing the best NCC. The utility of the method is evident in the second image: it improves the signal-to-noise ratio and reveals slight diffraction bands. Although it is insufficient for conventional Hough transform indexing, dictionary indexing succeeds. Using this method guarantees a solution, even if incorrect, so only data points with an NCC above 0.12 were considered correctly indexed and subsequently retained. Consequently, this EBSD map indexing exceeded a rate of 95%, facilitating a proper grain size distribution analysis for the various samples. Figure 4 illustrates the difference between Hough and dictionary indexing with NLPAR application for the B4-F5 sample (crushed for 4 h and sintered for 5 min), which exhibited the finest microstructure.

## 3. Material Characterization

Samarium cobalt exists in several crystalline phases. In this study, we focused on the SmCo_5_ phase which exhibits the most promising properties in terms of coercivity. A SEM image of the powder is shown in Figure 5. The powder features a coarse particle size distribution and significantly heterogeneous particle sizes.

Figure 6 presents the XRD pattern of the powder as received from the supplier, alongside those of the crushed powders for comparison. We observe, as expected, that the main phase is SmCo_5_, with the Sm_2_Co_7_ phase also present as well as some residual metallic Sm. The powder is super-stoichiometric in terms of samarium in order to, according to the literature, aid sintering and compensate for samarium evaporation during thermal processing [18,19,26]. The addition of Sm can increase the coercivity [27]. Additionally, the diffraction peaks broaden with increased milling time, indicating a reduction in crystallites size.

The XRD patterns of the densified samples are presented in Figure 7. After sintering, the formation of the SmCo 2:17 and Sm_2_Co_3_ phases is observed. Despite the low oxygen partial pressure in the SPS chamber, slight oxidation of samarium occurred during sintering, consuming the metallic samarium initially present in the starting powder. This oxidation led to the formation of samarium oxides, resulting in a sub-stoichiometric samarium phase, SmCo 2:17. Since samarium oxide is non-magnetic, its presence can adversely affect the magnetization properties of the final compound. The densities of the sintered samples ranged from 8.35 to 8.50 ± 0.05 g/cm^3^.

## 4. Microstructure Analysis

Figure 8 shows EBSD maps of the five samples in inverse pole figure along the out-of-plane direction (IPFZ). The unground sample displays a highly heterogeneous and coarse microstructure, featuring very large grains measuring more than a hundred micrometers alongside regions of small micrometric grains. This sample serves as a reference state for comparison with the three other samples. They all exhibit homogeneous microstructures with nanometric grain sizes following a lognormal distribution. Figure 9 displays three maps, with each grain colored according to its equivalent diameter, along with the fit of their distributions following a lognormal law. Table 1 provides results of the grain size analysis expressed in equivalent diameter, presenting not only the arithmetic mean and largest grain size but also the confidence intervals at [5%; 50%; 95%], i.e., [exp(μ − 2∙σ); exp(μ); exp(μ − 2∙σ)], where μ and σ represent the mean and standard deviation of the normal distribution fitted on the natural logarithm of grain size. The grinding of raw powder not only substantially reduced the grain size in the sintered materials’ microstructures but also promoted a more uniform grain size distribution. Notably, the two samples ground for 4 h have finer grain sizes than the one ground for 2 h. Amongst the two samples ground for 4 h, the one with a longer sintering time displays slightly larger grain sizes, attributed to grain growth during the thermal treatment.

## 5. Magnetic Properties

The magnetic measurements of the different powders (Figure 10) reveal the impact of grinding on the shape of the hysteresis loops and on the coercive field values. It is clearly evidenced that the crushed powders exhibit higher coercive field values compared to the raw powder. However, as the grinding time increases, the Hc value progressively decreases. This trend can be explained by the mechanism illustrated in Figure 1. With prolonged grinding, the particle size is reduced to below the size of a magnetic monodomain, resulting in a decrease in the coercive field. The hysteresis loops of the powders ground for 1 h and 2 h exhibit a “wasp-waist” shape, which may be attributed to their heterogeneous particle size distributions with varying coercivities [28]. The presence of multiple magnetic phases can lead to a pinning effect that blocks the reversal of a certain magnetic moment during magnetization or demagnetization. This phenomenon can cause the presentation of hysteresis loops in the form of “wasp-waisted” waves [29]. When grinding is sufficiently long and energetic to significantly reduce the size of the crystallites, this effect appears to be less pronounced, as seen in samples ground for 4 h. As grinding time increases (i.e., 4 h), a homogenization of particle sizes is observed. The coercive field (Hc) value for the raw powder, initially 1407 Oe, increases to 9305 Oe, 7275 Oe and 2087 Oe for the powders ground for 1 h, 2 h and 4 h respectively. Since the maximum specific energy density (BH)_max_ depends on the coercive field, the highest value obtained for the powders—23.5 kJ/m^3^—is observed for the powder ground for 1 h.

After sintering, a noticeable widening of the hysteresis loops is observed in Figure 11. All samples obtained from sintered crushed powders exhibit significantly higher coercive field values compared to those sintered from the raw powder (Table 2). This phenomenon is likely due to the reduction in crystallite size after grinding, falling below the size of a single magnetic domain. The size of the crystallites measured by EBSD is associated with the grain size. During the sintering cycle, grain growth occurs, bringing the particle size closer to that of a monodomain, which in turn affects the coercive field (Hc) values. In particular, the coercive field is highest for the samples sintered with powders grounded for the longest duration (i.e., 4 h). The homogenization of the size distribution, combined with the grain growth during sintering, results in a specimen with a coercive field (Hc) of 18,841 Oe and a maximum energy product ((BH)_max_) of 53.7 kJ/m^3^, as measured with the SQUID with a magnetic field of 9 T. However, after 20 min of dwell time at 700 °C, a decrease in the coercive field is observed (see Figure 11), compared to a dwell time of 5 min. This can be attributed to a slight grain growth or a slight increase in the samarium oxide phase, which increases microstructural heterogeneity and facilitates magnetization reversal (see Figure 9). An increase in the M_r_/M_s_ ratio is also observed when measuring compacts produced from milled powders. The particles are smaller in size, which affects the squareness factor by increasing the M_r_ value. The values reported for the B0-F5, B1-F5, B2-F5 and B4-F20 samples are determined from measurements taken with a VSM under an external field of 3 T. As saturation is not achieved, the reported values re underestimated.

## 6. Discussion

Grinding reduces crystallite size in the SmCo_5_ powder. This is clearly evidenced by the broadening of the XRD peaks, which confirms the effectiveness of the process. Hysteresis loop measurements on the powders suggest that grinding increases the coercive field. However, samples subjected to prolongated grinding exhibit a decrease in Hc values, likely due to their particle size falling below the monodomain threshold. After sintering, grain growth enables higher Hc values to be achieved. Once densified, samples ground for longer durations demonstrate both a higher coercive field and an improved maximum energy product ((BH)max). After SPS, all samples contain trace amount of Sm_2_O_3_ and the Sm_2_Co_17_ phase. Despite the low oxygen partial pressure inside the SPS chamber, the residual oxygen is sufficient to oxidize samarium, resulting in the formation of the Sm-deficient Sm_2_Co_17_ phase. While, this phase exhibits higher saturation magnetization than the SmCo_5_ phase, its coercive field is lower. There appears to be no direct correlation between the formation of these phases and the grinding process. There is a clear relationship between the grinding time and the magnetization values of the different samples. EBSD analyses reveal a significant reduction in grain size with increased grinding time. Using the NLPAR method and image correlation, indexing rates of approximatively 96% can be achieved. Longer grinding times seem to promote a more homogeneous size distribution. Additionally, combining a longer grinding time with shorter sintering cycles appears to yield a more uniform particle size distribution. For instance, when the grain sizes are close to 50 nm, as in the sample ground for 4 h, it is possible to reach, with a dwell time of 5 min, a coercive field of 18,841 Oe and a (BH)max of 53.7 kJ/m^3^.

## 7. Conclusions

Optimization of the grinding process of SmCo5 magnet powder, followed by densification via SPS under a vacuum, yielded SmCo magnets exhibiting very high coercive field values of the order of 18,800 Oe. The determined (BH)_max_ value remains lower than those reported in the literature for SmCo_5_ (between 135 and 172 kJ/m^3^) [11,30]. This difference can be explained by the oxidation of samarium during sintering. The presence of multiple phases generates areas sensitive to magnetization reversal, which has the effect of decreasing the Hc value. Our findings indicate that the coercive field of the sintered samples increases with grinding time. EBSD image analysis, enhanced by the NLPAR (Non-Local Pattern Averaging Reconstruction) method and image correlation techniques, played a role in precisely characterizing the grain size distribution (centered around 50 nm). These insights pave the way for achieving magnets with more open and rectangular hysteresis loops, as well as higher remanent magnetization, particularly by using field-oriented powders prior to sintering. By aligning the easy magnetization axes of the powders, it would be possible to further increase the measured power densities.

## Figures and Tables

**Figure 1:**
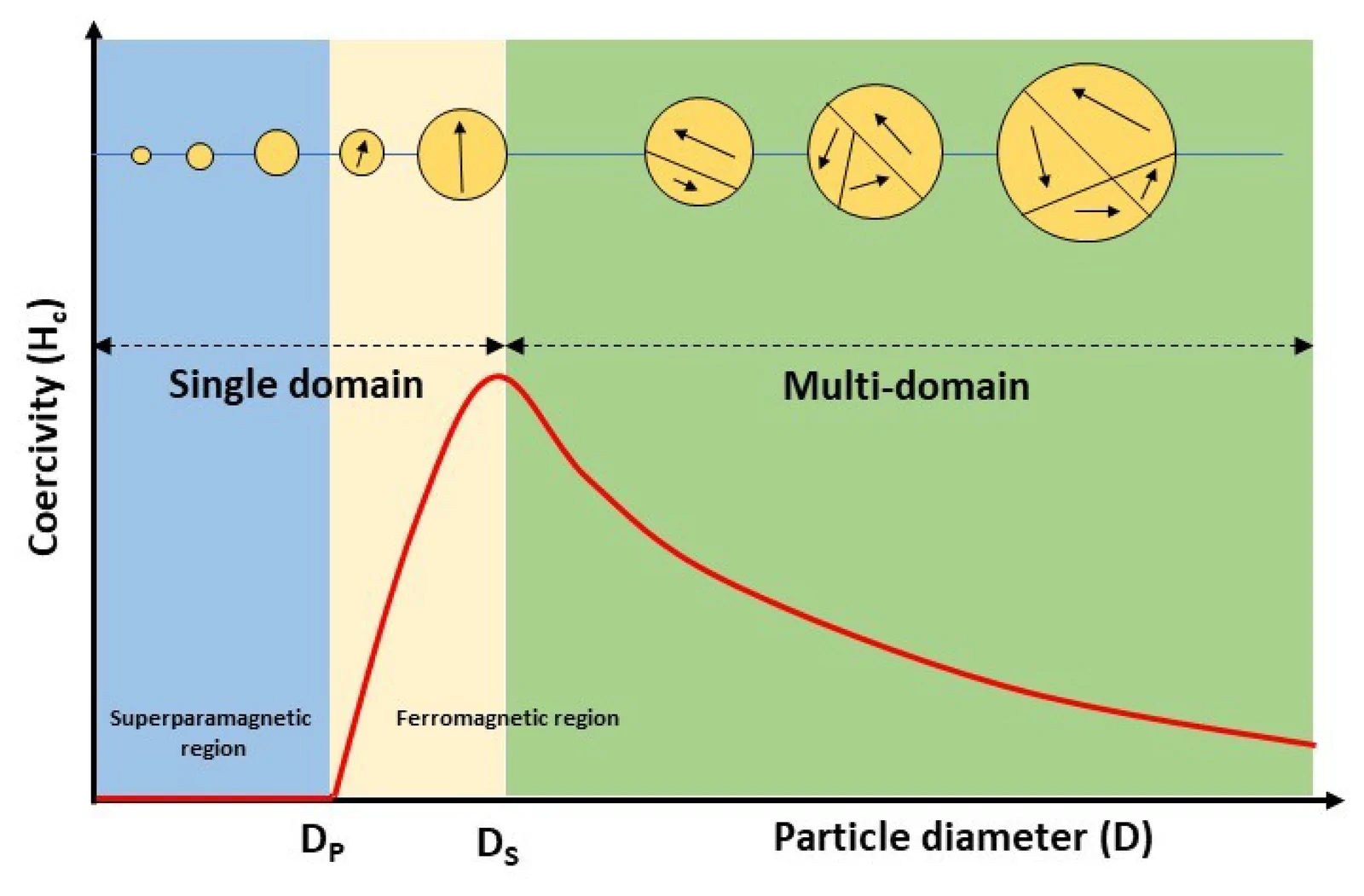
A schematic showing the coercivity (Hc) behavior in red line and magnetic domain (yellow spheres) with the magnetic moment represented by arrows of a magnetic particle as a function of D (diameter), Adapted from Ref. [14].

**Figure 2:**
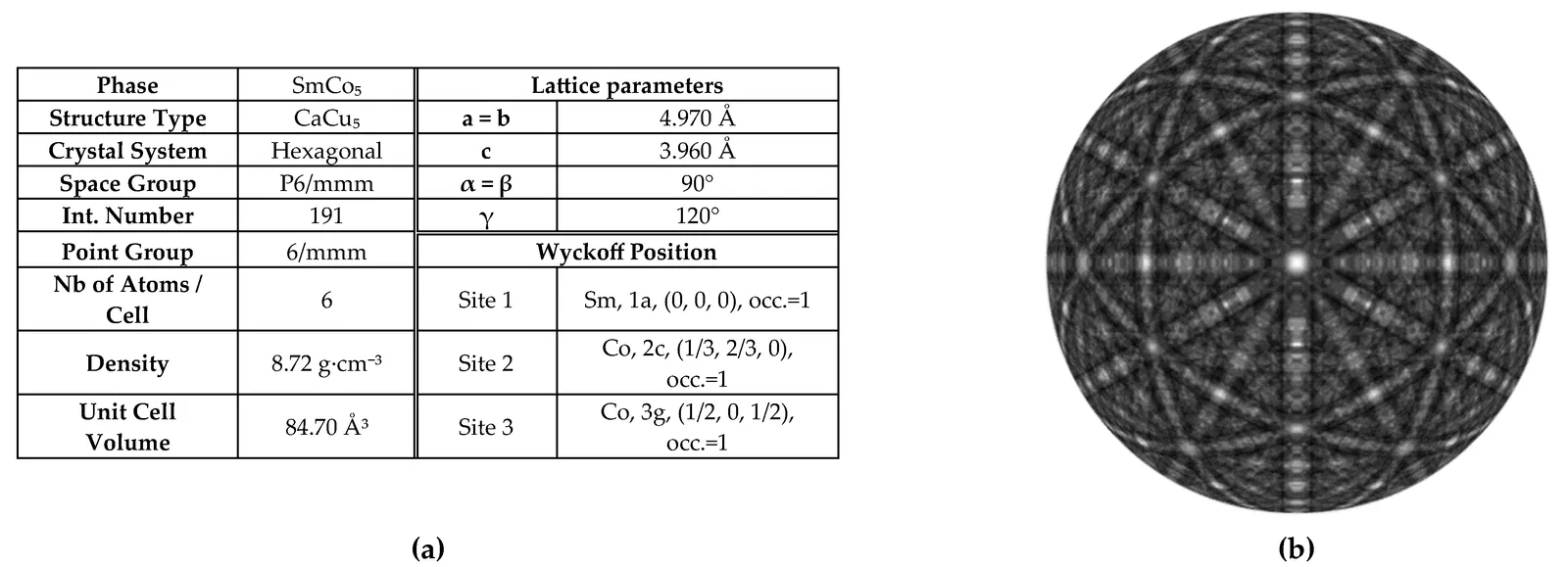
(**a**) Crystallographic data of the SmCo_5_ crystal, from [24], and (**b**) Simulated master pattern of electron diffraction of this crystal under a 15 kV beam visualized on a 3D sphere.

**Figure 3:**
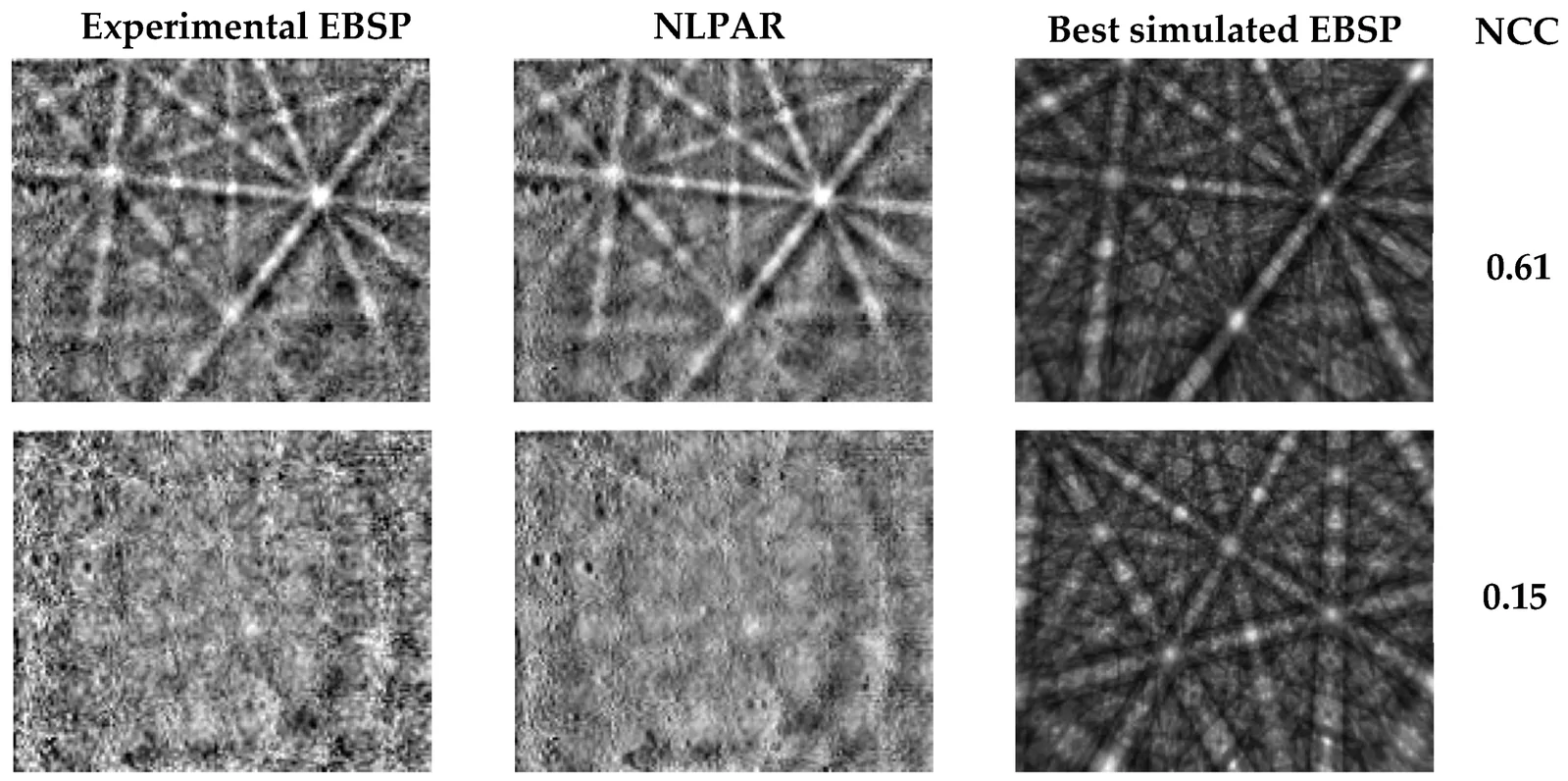
Experimental EBSP, NLPAR average, and simulated pattern showing the highest similarity and NCC value for (**top**) a good signal pattern and (**bottom**) a pattern with a poor diffracting signal.

**Figure 4:**
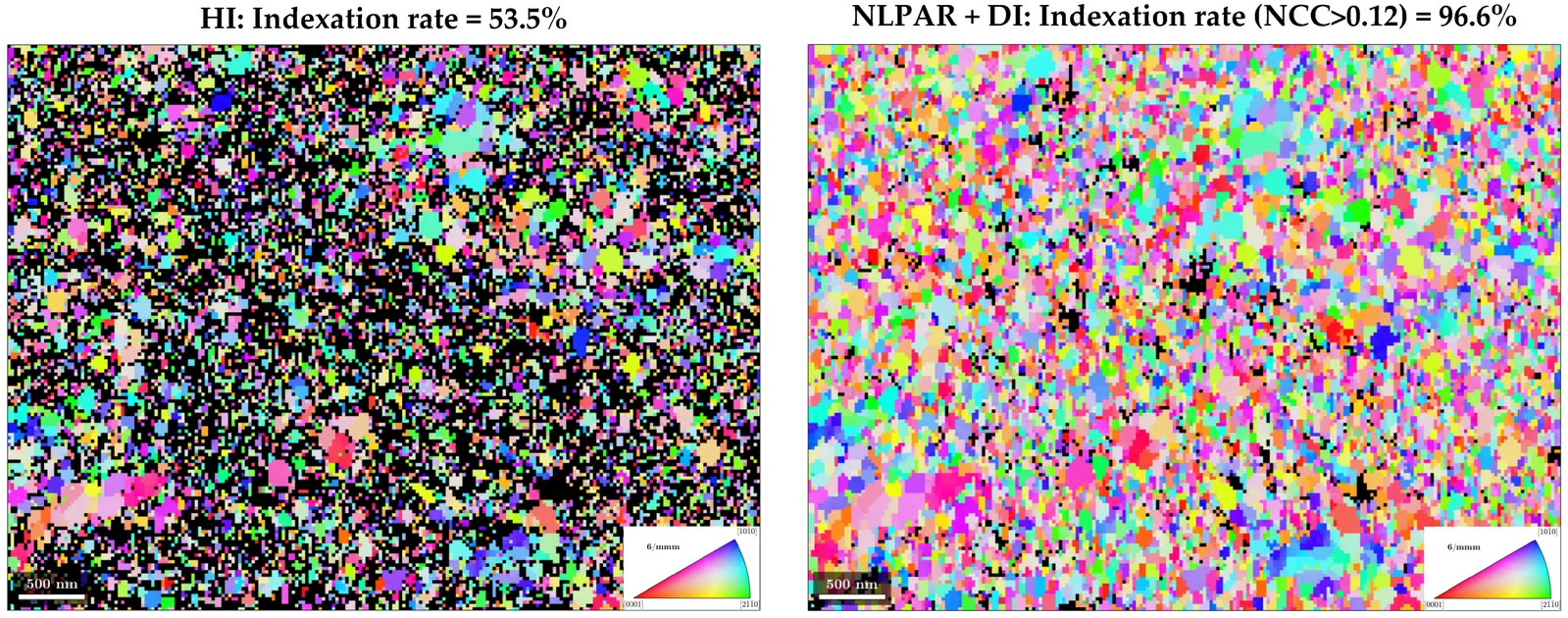
IPFZ maps of sample B4-F5 (**left**) after indexing by the Hough transform method and (**right**) after dictionary indexing following the average pattern by the NLPAR method.

**Figure 5:**
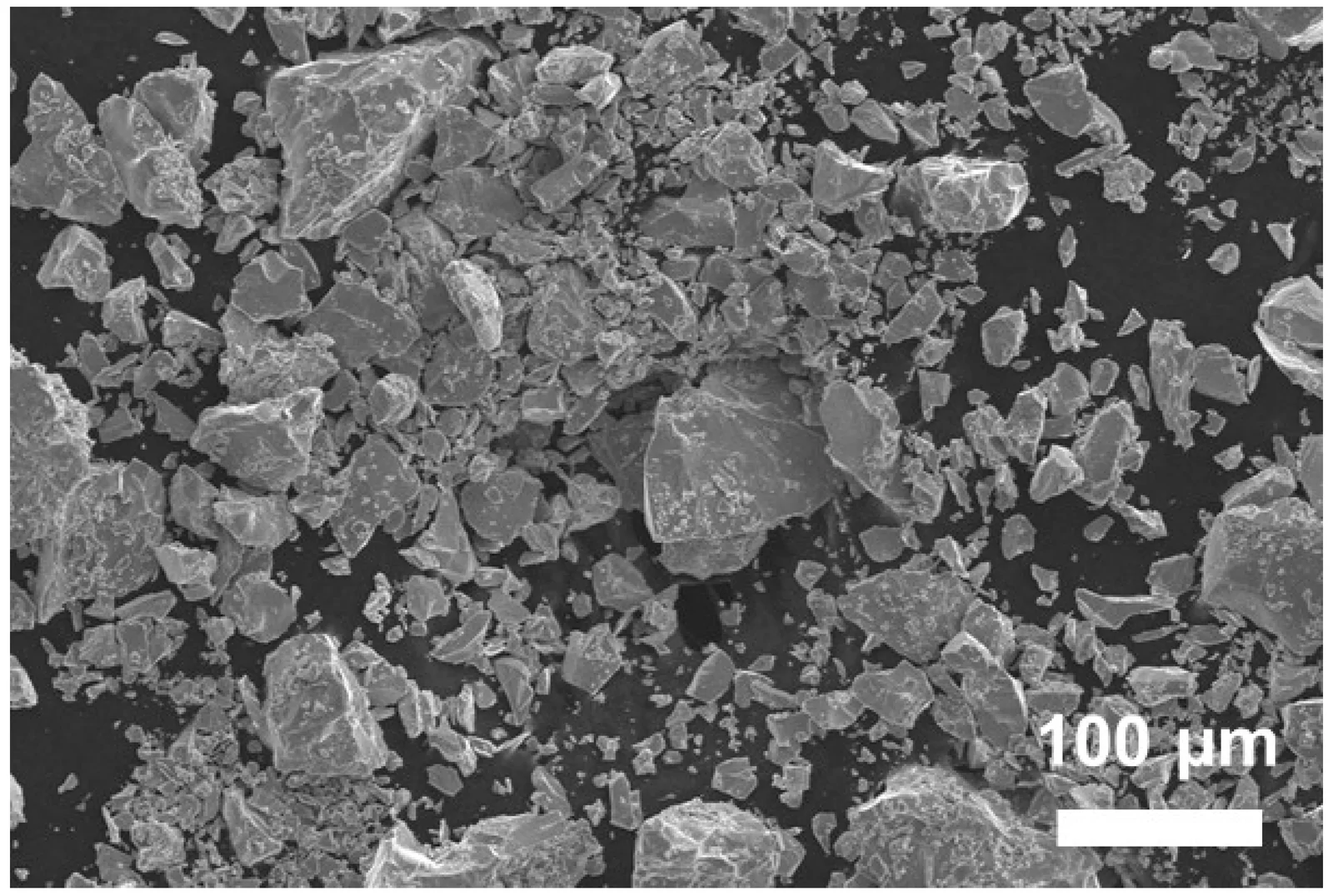
SEM image of the raw SmCo_5_ powder.

**Figure 6:**
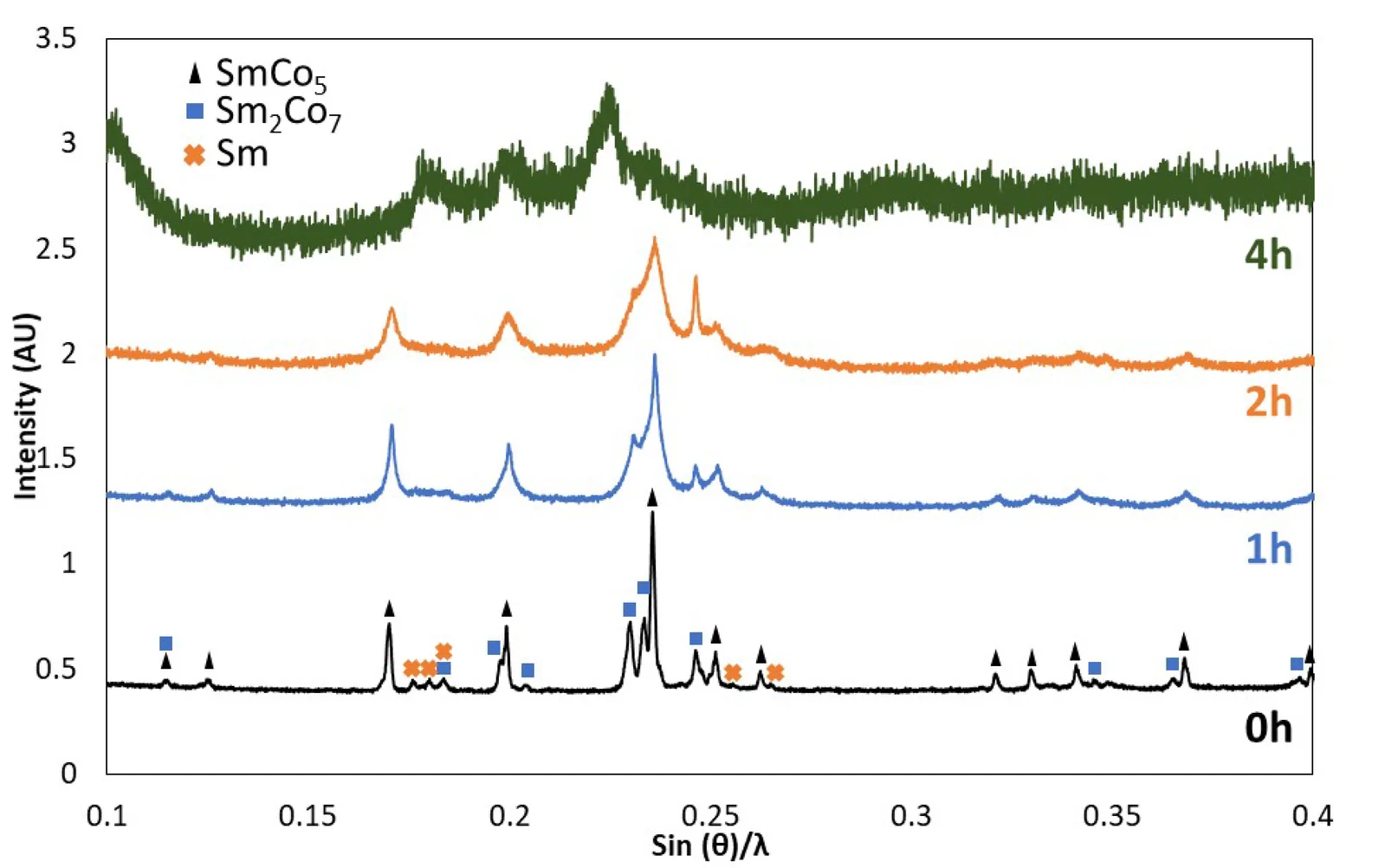
XRD of the powder after 0 h, 1 h, 2 h, and 4 h of grinding under argon.

**Figure 7:**
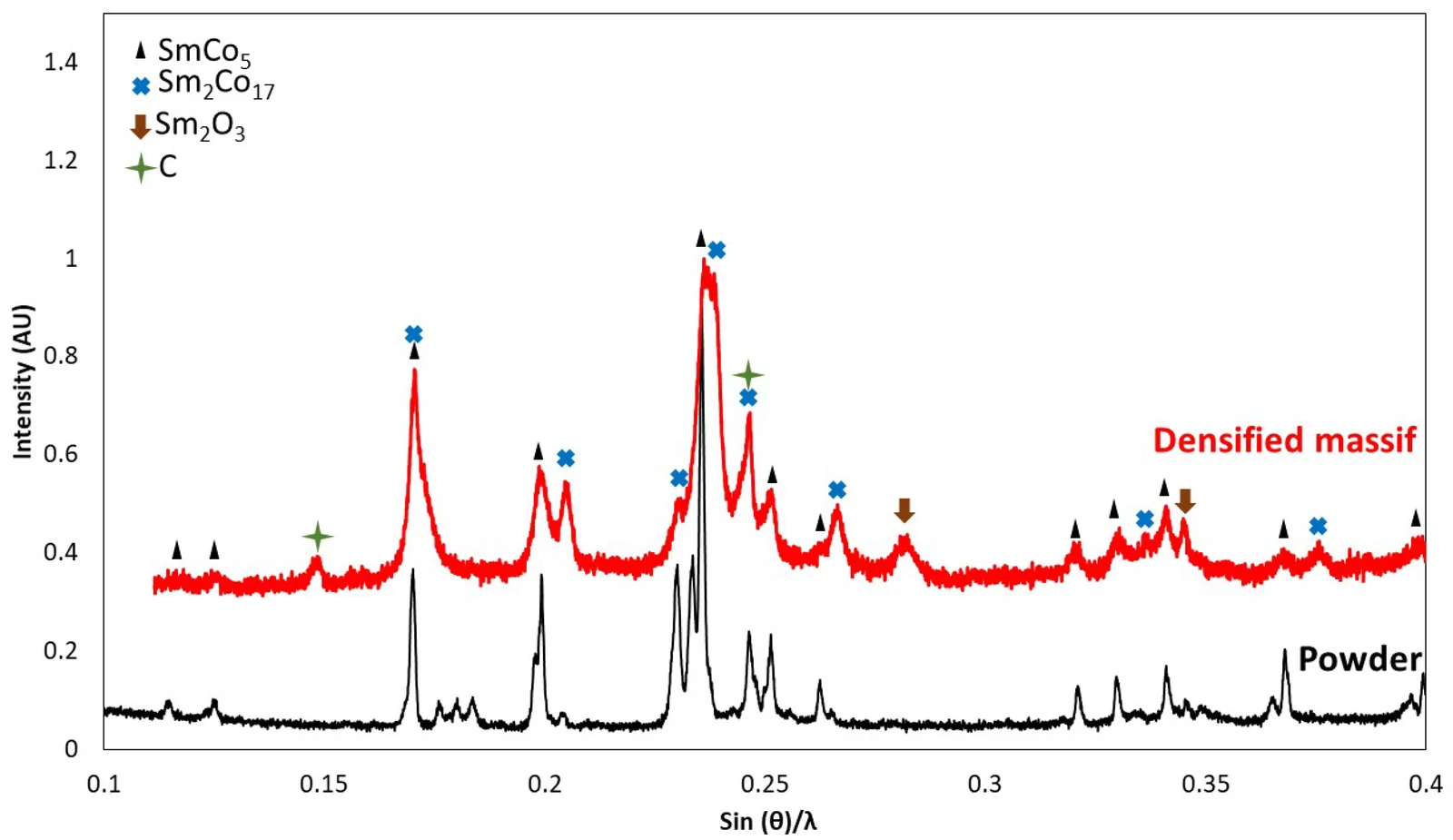
XRD of the powder and a bulk sample densified by SPS.

**Figure 8:**
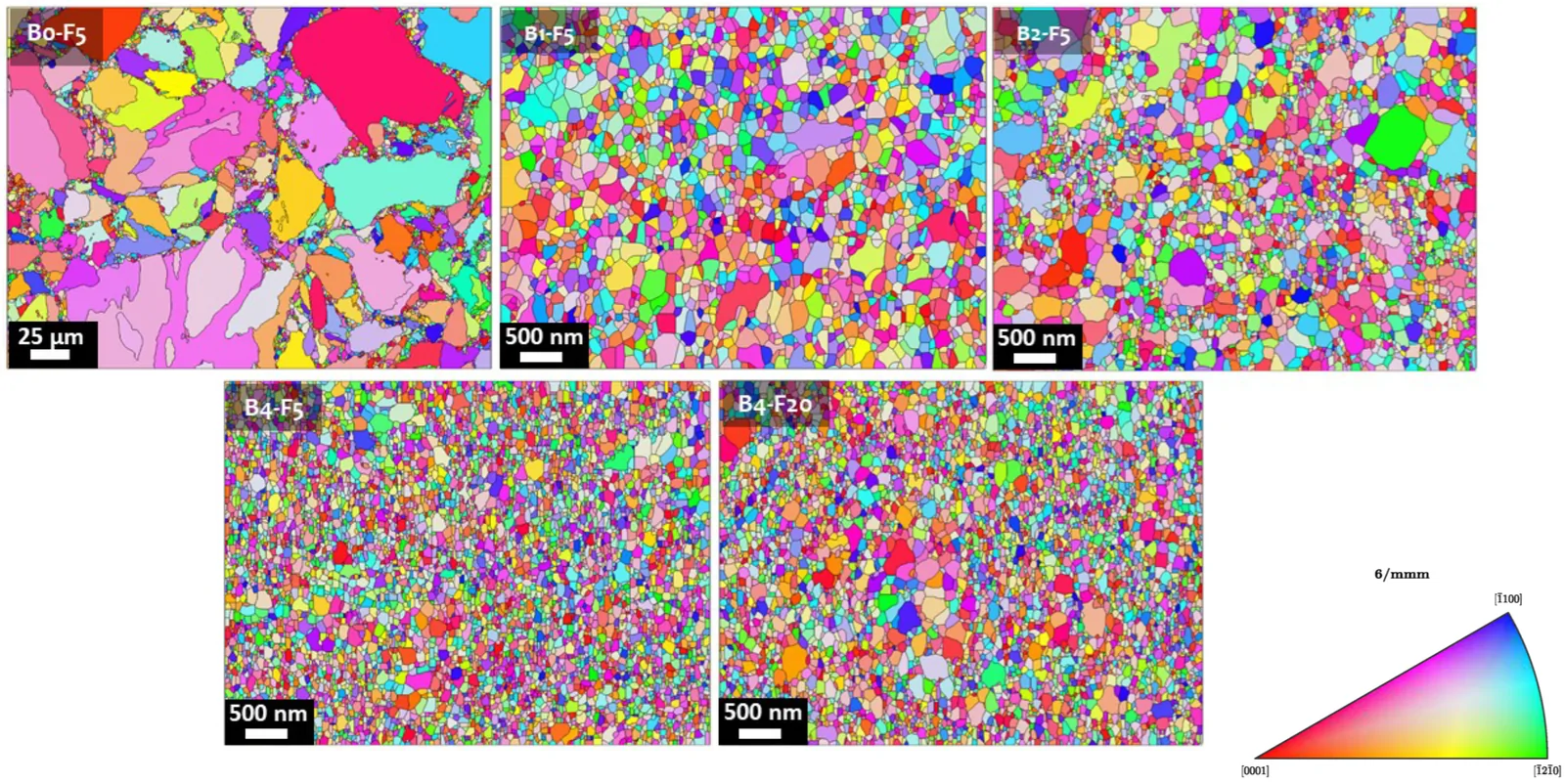
IPFZ mappings of the five densified samples depending of the grinding. Note the very different scale of the NB (without grinding) sample compared to the other four.

**Figure 9:**
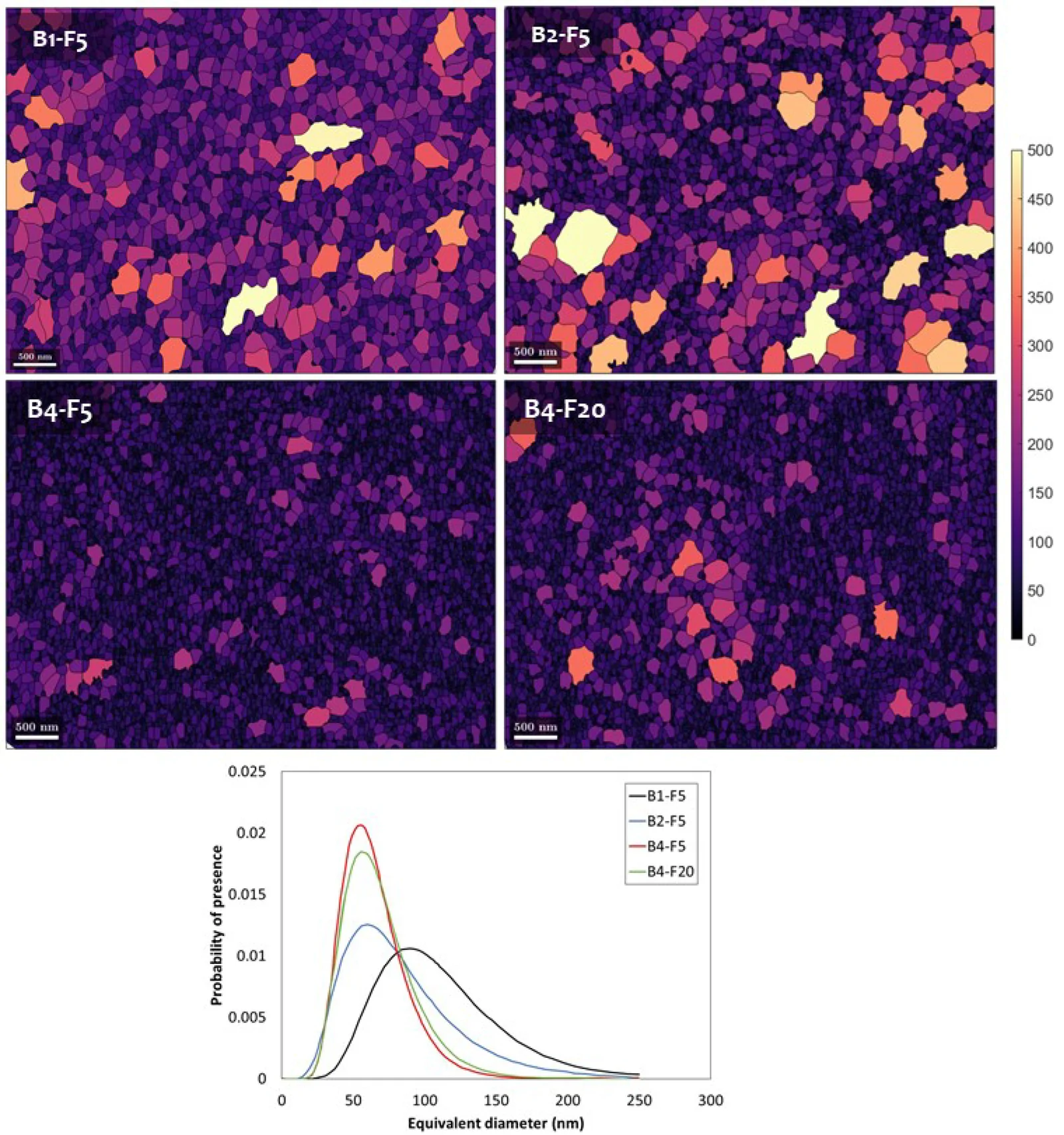
Grain size mappings of the four milled and sintered samples (equivalent diameter in nm) and their lognormal distribution.

**Figure 10:**
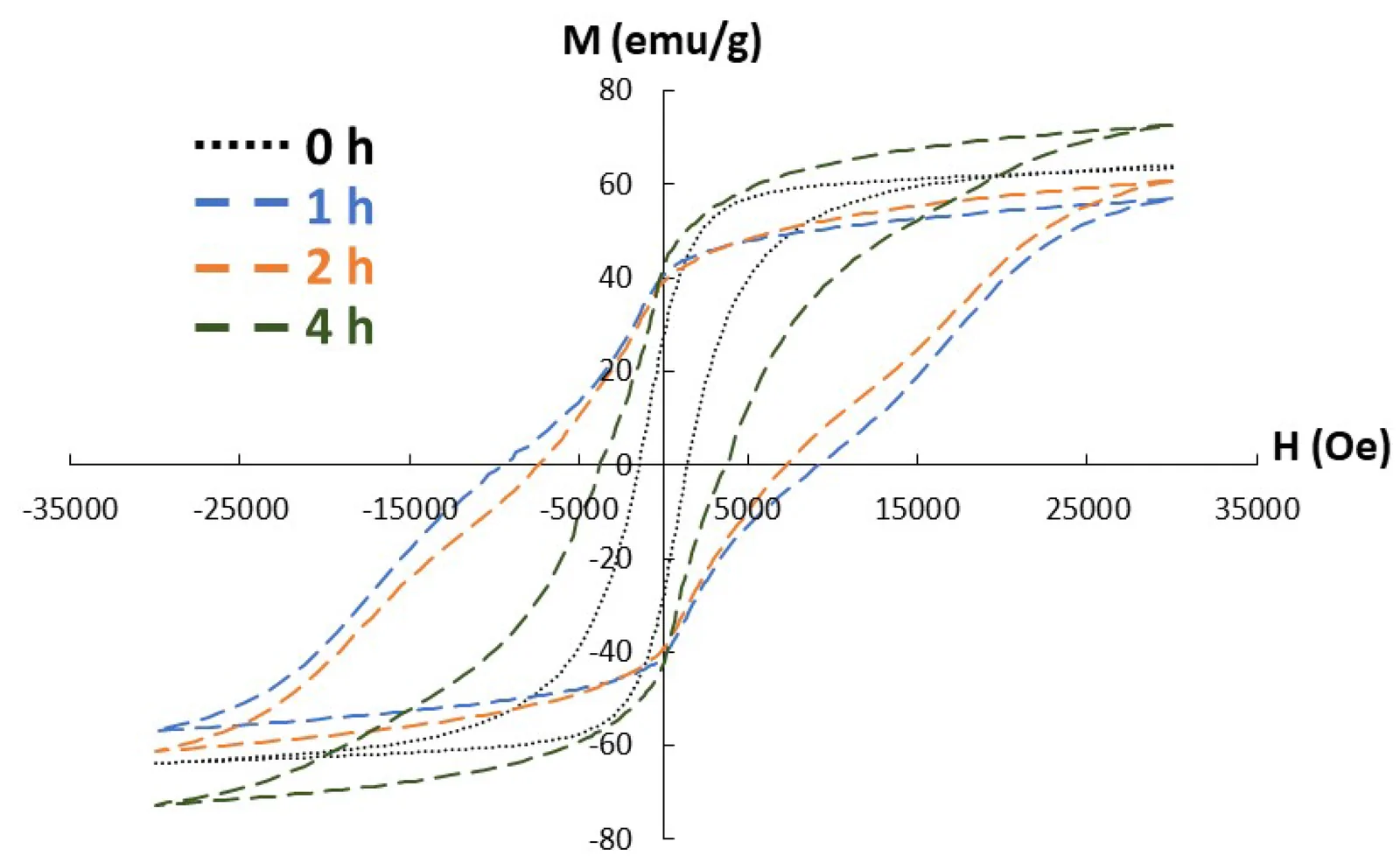
Hysteresis loops of powders ground for 0 h, 1 h, 2 h and 4 h.

**Figure 11:**
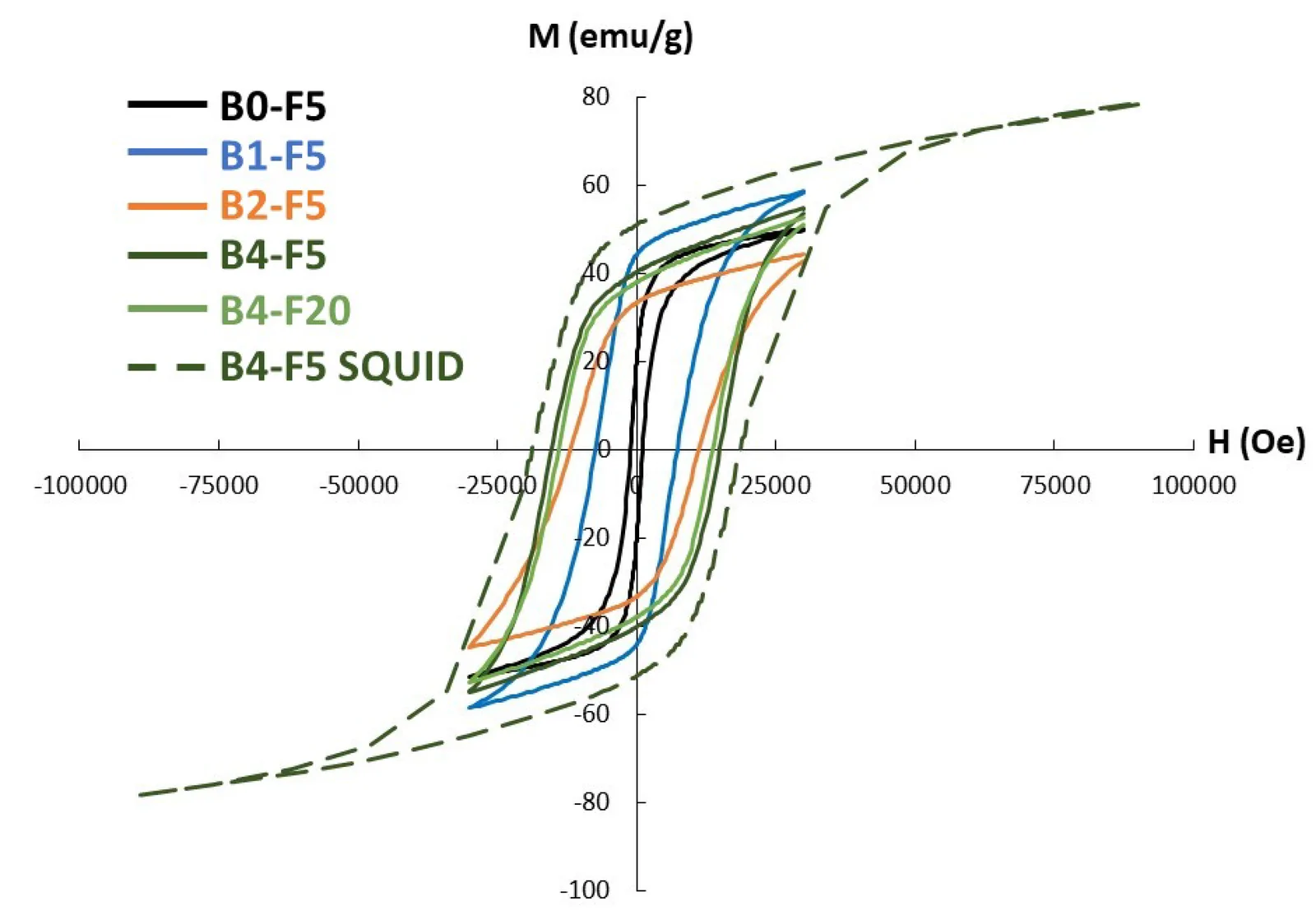
Hysteresis loops of samples sintered from ground powder.

**Table 1:** Summary of the grain size distribution analysis of the four milled and sintered samples (equivalent diameter).

Sample	Arithmetic Mean	Larger Grain	Lognormal Fit: 5%/50%/95%		
			Exp (μ − 2σ)	Exp (μ)	Exp (μ + 2σ)
B1-F5	112 nm	510 nm	30 nm	104 nm	226 nm
B2-F5	84 nm	684 nm	25 nm	72 nm	209 nm
B4-F5	64 nm	278 nm	27 nm	59 nm	128 nm
B4-F20	68 nm	348 nm	27 nm	62 nm	142 nm

**Table 2:** Values of the coercive field Hc, the maximum specific energy density (BH)_max_, the remanent magnetization and the squareness ratio of samples sintered from ground powders.

Samples	Hc (Oe)	(BH)_max_ (kJ/m^3^)	M_r_ (emu/g)	M_r_/M_s_	External Magnetic Field (Tesla)
B0-F5	1110	3.2	20.1	0.35	3
B1-F5	7417	34.0	44.2	0.59	3
B2-F5	11,523	22.7	33.3	0.49	3
B4-F5	15,117	33.3	40.2	0.48	3
B4-F20	14,099	29.9	37.9	0.47	3
B4-F20 SQUID	18,841	53.7	51.5	0.55	9

## Data Availability

The original contributions presented in this study are included in the article. Further inquiries can be directed to the corresponding author.
